# Burden and patient characteristics associated with repeat consultation for unscheduled care within 30 days in primary care: a retrospective case control study with implications for aging and public health

**DOI:** 10.3389/fpubh.2023.1079755

**Published:** 2023-07-25

**Authors:** Valentin Richard, Leila Bouazzi, Clément Richard, Stéphane Sanchez

**Affiliations:** ^1^General Practice Department, University of Tours, Tours, France; ^2^University Committee of Resources for Research in Health (CURRS), University of Reims, Marne, France; ^3^SOS Medecin, Troyes, France; ^4^Public Health and Performance Department, Champagne Sud Hospital, Troyes Hospital, Troyes, France

**Keywords:** quality of care, general practice, duty period, primary care supress (MeSH), public health

## Abstract

**Introduction:**

Repeated consultations in primary care represent a significant burden on healthcare services. Characterizing the patients who repeatedly attend ambulatory care would enhance our understanding of the healthcare needs of this population, with a view to providing appropriate services. The aim of this study was therefore to identify the factors associated with repeated consultation in unscheduled care. Our secondary aim was to explore the specific profile of patients aged >65 years.

**Methods:**

A retrospective case–control study comparing re-consultation within 30 days at a primary care facility versus non-reconsulting patients, defined as those who did not reconsult within 30 days, among patients consulting over a period of 1 year (1 January to 31 December 2019). Data was collected for a random sample of 5,059 consultations. Patients and controls were matched for age ± 5 years, and sex.

**Results:**

The main factors associated with repeat consultation were an initial consultation late at night (midnight to 6.00 am; OR 1.31, 95%CI 1.20–1.44), and psychological disorders as the main diagnosis (OR 1.33, 95%CI 1.20–1.48). Conversely, consulting at the weekend was associated with a lower likelihood of repeat consultation (OR 0.82, 95% 0.85–0.91).

**Conclusion:**

30-day reconsultations were significantly more frequent after late night consultation. This could be used as an indicator of the quality of care to assess performance of general practice teams with implications for improving overall health of an aging population.

## 1. Introduction

Repeated consultations in primary care represent a significant burden on healthcare services. Evidence suggests that the 3% of patients who consult most frequently account for 15% of the clinical consultation time of general practitioners (GPs) (1). Definitions of these patient groups have been developed in primary care, in emergency departments and in the hospital setting (2). The characteristics of frequent attenders have also been widely studied, and both female sex and age > 65 years have been shown to be associated with repeated healthcare consultations (3, 4). Certain diseases are also more common among frequent attenders, especially chronic diseases, such as hypertension and type 2 diabetes mellitus (5), but also psychiatric and musculo-skeletal disorders (3). Socio-demographic characteristics as well as the individual’s subjective perception of their own health are also associated with an increased likelihood of frequent consultations (6, 7). Finally, an analysis of the prescriptions issued during these repeat consultations showed that there is an increased frequency of prescription, for longer durations, and for more expensive drugs (8).

Studies to date have been performed in patients consulting in primary care, over long periods of time (1, 3). However, data are less abundant concerning the use of unscheduled care in the shorter term, and existing works have focused on specific diseases (9, 10). The frequency of repeat consultations at unscheduled care services, as well as the motives and characteristics of the patients, are not well documented. Yet, attendance at unscheduled care services is constantly rising, with an increase of 38% documented over the last 10 years by *SOS Médecins France*, a nationwide service providing round-the-clock healthcare services (11). Studies investigating out-of-hours services in primary care report conflicting results regarding admissions to the emergency department and unscheduled primary care during off-hours (12).

In the Aube Department of Eastern France, the medical demographic is below the national average, with a large part of healthcare delivery still being carried out by general practitioners (GPs) who have reached retirement age but continue to practice (13). The downward trend in healthcare accessibility in this Department is likely to continue, with a projected decrease in the number of practicing GPs of 10% by 2030 (14). Therefore, in contexts where there is a dearth of primary care practitioners, studying the characteristics of frequent primary care attenders, who are potentially resource- and time-consuming, could help to better adapt public health policy and the organization of care delivery. It may also identify areas that are congruent or discordant with the risk factors of frequent attendance identified in scheduled care (3–6). This could ultimately help to define quality criteria for the organization of unscheduled care services in geographical areas with a below-average level of primary care services. The aim of this study was therefore to identify the factors associated with repeated consultation in unscheduled care in the Aube Department of France. Our secondary aims was to explore the specific profile of patients aged 65 years with repeated consultations.

## 2. Materials and methods

### 2.1. Study design and population

We performed a single-center case–control study, comparing patients who returned for a repeat consultation within 30 days after an initial visit (“cases”), versus patients who did not repeat-consult within 30 days (“controls”), at an unscheduled care service run by *SOS Médecins* in the Aube Department, France. A consultation was defined as any contact between a physician from the *SOS Médecins* unscheduled care service, and a patient, be it during a home-visit, at the surgery, or by teleconsultation.

The study population was composed of patients consulting the *SOS Médecins* unscheduled care service between 1 January and 31 December 2019.

Inclusion criteria were age > 18 and <75 years, and absence of explicit opposition to the use of routinely collected data for the purposes of medical research. Using the database provided by SOMENOR, the company in charge of data management for *SOS Médecins*, we retrieved the data for a random sample of consultations from the period from 1 January to 31 December 2019. The population of patients between 18 and 75 years of age comprised 27,431 consultations during this period. We used a random sample of 5,061 consultations (18% of the sample size), of which 59 were excluded due to missing data for the main outcomes. For each case, we randomly selected controls from among the 3,785 patients who did not re-consult within 30 days after an initial consultation during the study period using an *ad hoc* matching algorithm. Then, each case (i.e., patient with repeat-consultation within 30 days) was matched with one control (i.e., a patient who did not return for repeat consultation within 30 days after the initial visit). Patients and controls were matched for age (±5 years) and sex.

**Figure 1:**
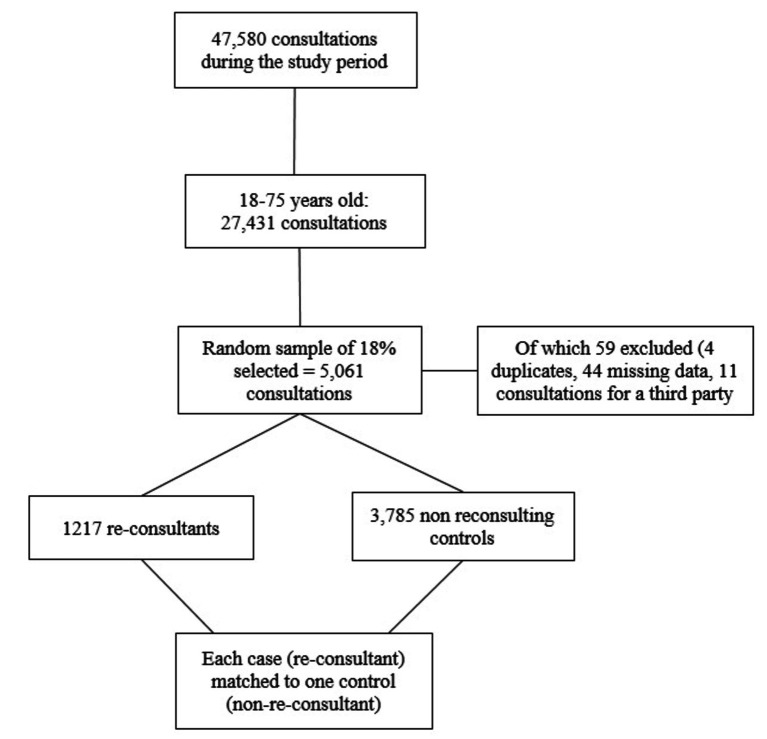
Flowchart of the study.

The flowchart of the study population is detailed in Figure 1.

### 2.2. Endpoints and variables recorded

The primary outcome was a repeat consultation with 30 days after an initial consultation with *SOS Médecins* unscheduled care services. Secondary endpoints were the time of day when the consultations took place; need for hospital admission; the diagnosis retained at each consultation.

Using the database provided by SOMENOR, the company in charge of data management for *SOS Médecins*, we retrieved the data for a random sample of consultations from the period from 1 January to 31 December2019.

For each consultation, we recorded the patient’s age and sex. Regarding the consultations, we recorded: triage category (when the patient calls the *SOS Médecins* unscheduled care service to ask for an appointment, the calls are categorized by degree of urgency, namely: non urgent, relative emergency, life-threatening emergency); reported motive for consulting; timing of the consultation (weekend, holiday, out-of-hours (8 pm to midnight, and 6.00 am to 8.00 am), late night (midnight to 6.00 am)). We also recorded the treatments and procedures prescribed, the diagnostic group retained, as per the International Classification of Primary Care (15), and whether or not a request for hospital admission resulted from the consultation.

### 2.3. Statistical analysis

Continuous variables are presented as mean ± standard deviation if normally distributed, and otherwise, as median and range. Discrete variables are presented as number (percentage). Each patient who re-consulted within 30 days after a first consultation at SOS Medecins unscheduled care services was matched to a control patient who did not re-consult within 30 days for age ±5 years, and sex, as described above.

Characteristics were compared between patients and controls using univariate generalized estimating equations (GEE; with a binomial distribution and logit function) for discrete variables, and for continuous variables, using a univariate mixed linear model, with adjustment for the matching variables as a random effect. Variables that yielded a *p*-value < 0.10 by univariate analysis were then included in a multivariate model. Results are presented as odds ratios (ORs) and 95% confidence intervals (CIs) to estimate effect size. The time to repeat consultation was defined as the time between discharge from the initial contact, and the start of the repeat consultation. Patients who did not re-consult within 30 days were censored. The median time to repeat consultation was compared according to patient characteristics using the log-rank test.

Factors significantly associated with repeat consultation (*p* < 0.10) were included in a Cox proportional hazards multivariate regression model with adjustment for the matching variables as random effects. Backward selection was applied with a threshold for exiting the model of 0.05. Results are reported as hazard ratios (HRs) and 95%CI. The proportional hazards assumption was verified by examining Schoenfeld residuals. Survival curves were drawn using the Kaplan Meier method. All statistical analyses were performed using SAS version 9.4 (SAS Institute Inc., Cary, NC) and a *p*-value <0.05 was considered statistically significant.

### 2.4. Ethical considerations

According to French legislation, the retrospective, observational design of this study, using routinely collected data from the medical files, did not require ethics committee approval (16). The study was performed in compliance with national legislation governing epidemiological research, the Helsinki Declaration and its amendments, and the principles of Good Clinical Practice. All patients were informed *via* posters in the waiting rooms that their data could be used for the purposes of medical research.

## 3. Results

From a total of 47,580 consultations that took place between 1 January and 31 December 2019, 27,431 were for patients within our target age range of 18 to 75 years. Among these, 5,061 (18.4%) were used for data collection, of which 59/5,061 (1.2%) were excluded due to missing data for the main outcomes. A final total of 5,002 consultations were included in the analysis: 3,785 single consultations, and 1,217 repeat consultations (Figure 1).

The characteristics of the 2 groups are presented in Table 1, before and after matching for age and sex. Before matching, comparison the 1,217 repeat consultations to the 3,785 single consultations showed a significant difference in patient age (*p* = 0.001), with repeat-consultation patients being younger. There was also a significantly higher proportion of women among those with repeat consultations (*p* = 0.008).

**Table 1:** Characteristics of the patients attending consultations in unscheduled primary care services (before and after matching on age and sex).

	Overall unmatched population			Matched population		
	Repeat consultation within 30 days			Repeat consultation within 30 days		
	Yes *N* = 1,217	No *N* = 3,785	*p*-value	Yes *N* = 1,217	No *N* = 1,217	*p*-value
Age, mean ± SD, years*	36.3 ± 14.2	37.9 ± 14.8	0.001	36.3 ± 14.2	36.3 ± 14.2	1.00
Sex*			0.008			1.00
Female	805 (66.1)	2,343 (61.9)		805 (66.1)	805 (66.1)	
Male	412 (33.9)	1,442 (38.1)		412 (33.9)	412 (33.9)	
Location of the consultation			0.01			0.08
Home	338 (27.8)	1,193 (31.5)		338 (27.8)	378 (31.1)	
At the surgery	879 (72.2)	2,591 (68.4)		879 (72.2)	839 (68.9)	
Time of the consultation						
Out of hours (6 pm-midnight, 6-8 am)	165 (13.6)	581 (15.3)	0.13	165 (13.6)	196 (16.1)	0.09
Late night (midnight to 6.00 am)	173 (14.2)	323 (8.5)	<0.0001	173 (14.2)	117 (9.6)	0.0001
Weekend	259 (21.3)	1,093 (28.9)	<0.0001	259 (21.3)	349 (28.7)	<0.0001
Motive for the consultation			0.10			0.09
Acute problem	1,201 (98.7)	3,755 (99.2)		1,201 (98.7)	1,208 (99.3)	
Chronic disease	16 (1.3)	30 (0.8)		16 (1.3)	9 (0.7)	
Triaged at first call	25 (2.0)	69 (1.8)	0.60	25 (2.0)	18 (1.5)	0.24
Main diagnosis†						
Blood, blood forming organs	1 (0.1)	4 (0.1)	1.00	1 (0.1)	2 (0.2)	0.62
Digestive	251 (20.6)	751 (19.8)	0.41	251 (20.6)	237 (19.5)	0.47
Ear/nose/throat	296 (24.3)	1,027 (27.1)	0.10	296 (24.3)	328 (27.0)	0.14
Circulatory system	17 (1.4)	103 (2.7)	0.01	17 (1.4)	35 (2.9)	0.03
Musculo-skeletal	205 (16.8)	568 (15.0)	0.08	205 (16.8)	190 (15.6)	0.40
Neurological	42 (3.4)	99 (2.6)	0.11	42 (3.4)	38 (3.1)	0.64
Psychological	105 (8.6)	202 (5.3)	<0.0001	105 (8.6)	55 (4.5)	<0.0001
Respiratory	107 (8.8)	408 (10.8)	0.07	107 (8.8)	124 (10.2)	0.25
Skin	56 (4.6)	206 (5.4)	0.30	56 (4.6)	65 (5.3)	0.42
Endocrine	4 (0.3)	14 (0.4)	0.86	4 (0.3)	7 (0.6)	0.42
Gynecological & urological	48 (3.9)	119 (3.1)	0.15	48 (3.9)	50 (4.1)	0.84
Social problems	84 (6.9)	284 (7.5)	0.57	84 (6.9)	86 (7.1)	0.87
Number of drugs prescribed, median [IQR]	3 [2–4]	3 [2–4]	0.29	3 [2–4]	3 [2–4]	0.29
Prescription of biology	108 (8.9)	372 (9.8)	0.33	108 (8.9)	116 (9.5)	0.58
Prescription of imaging	49 (4.0)	139 (3.7)	0.57	49 (4.0)	50 (4.1)	0.92
Hospitalisation	16 (1.3)	62 (1.6)	0.43	16 (1.3)	21 (1.7)	0.44

SD, standard deviation, IQR, interquartile range.

* Matching variables.

† Some categories were grouped to avoid groups with insufficient sample sizes.

In the unmatched population, among the patients with repeat consultations, the rate of consultation at the surgery was significantly higher than among patients with no repeat consultation (72.2% vs. 68.4% respectively). There was also a significant difference in the timing of the initial consultations between those who re-consulted and those who did not, with 14.2% those with repeat consultations having initially consulted late at night (from midnight to 6.00 am), versus only 8.5% of those who did not re-consult. Conversely, single consultations more frequently took place at the weekend (28.7%) compared to repeat consultations (21.3%; *p* < 0.0001).

Before matching, among the 12 diagnostic categories retained, there was a higher frequency of psychological disorders among patients with repeat consultations (8.6% vs. 5.3%, p < 0.0001). Conversely, cardiovascular disorders were more frequent among single consulters (2.7%) than in those with repeat consultations (1.4%). There was no significant difference in terms of the rate of prescription of biology tests or complementary examinations, or in the number of hospitalizations between groups.

After matching for age and sex, the significant differences between groups persisted, except for the location of the consultation, which was no longer statistically significant (Table 1).

Table 2 presents the factors independently associated with repeat consultation by multivariable analysis. The main factors associated with repeat consultation were an initial consultation late at night (midnight to 6.00 am; OR 1.31, 95%CI 1.20–1.44), and psychological disorders as the main diagnosis (OR 1.33). Conversely, consulting at the weekend was associated with a lower likelihood of repeat consultation (OR 0.82, 95% 0.85–0.91). Similarly, acute motives for consultation were also associated with a lower likelihood of repeat consultation (OR 0.76, 95%CI 0.61–0.96).

**Table 2:** Factors associated with repeat consultation within 30 days in the matched population.

	Repeat consultation within 30 days			
	Yes *N* = 1,217	No *N* = 1,217	OR (95%CI)	*p*-value
Location of the consultation				0.002
Home	338 (27.8)	378 (31.1)	1.00 (réf)	
At the surgery	879 (72.2)	839 (68.9)	1.15 (1.05–1.26)	
Time of the consultation				
Out of hours (6 pm-midnight, 6-8 am)	165 (13.6)	196 (16.1)	0.94 (0.84–1.06)	0.35
Late night (midnight to 6.00 am)	173 (14.2)	117 (9.6)	1.31 (1.20–1.44)	<0.0001
Weekend	259 (21.3)	349 (28.7)	0.82 (0.75–0.91)	0.0002
Motive for the consultation				
Acute	1,201 (98.7)	1,208 (99.3)	0.76 (0.61–0.96)	0.02
Main diagnosis				
Circulatory system	17 (1.4)	35 (2.9)	0.70 (0.48–1.03)	0.07
Psychological	105 (8.6)	55 (4.5)	1.33 (1.20–1.48)	<0.0001

**Figure 2:**
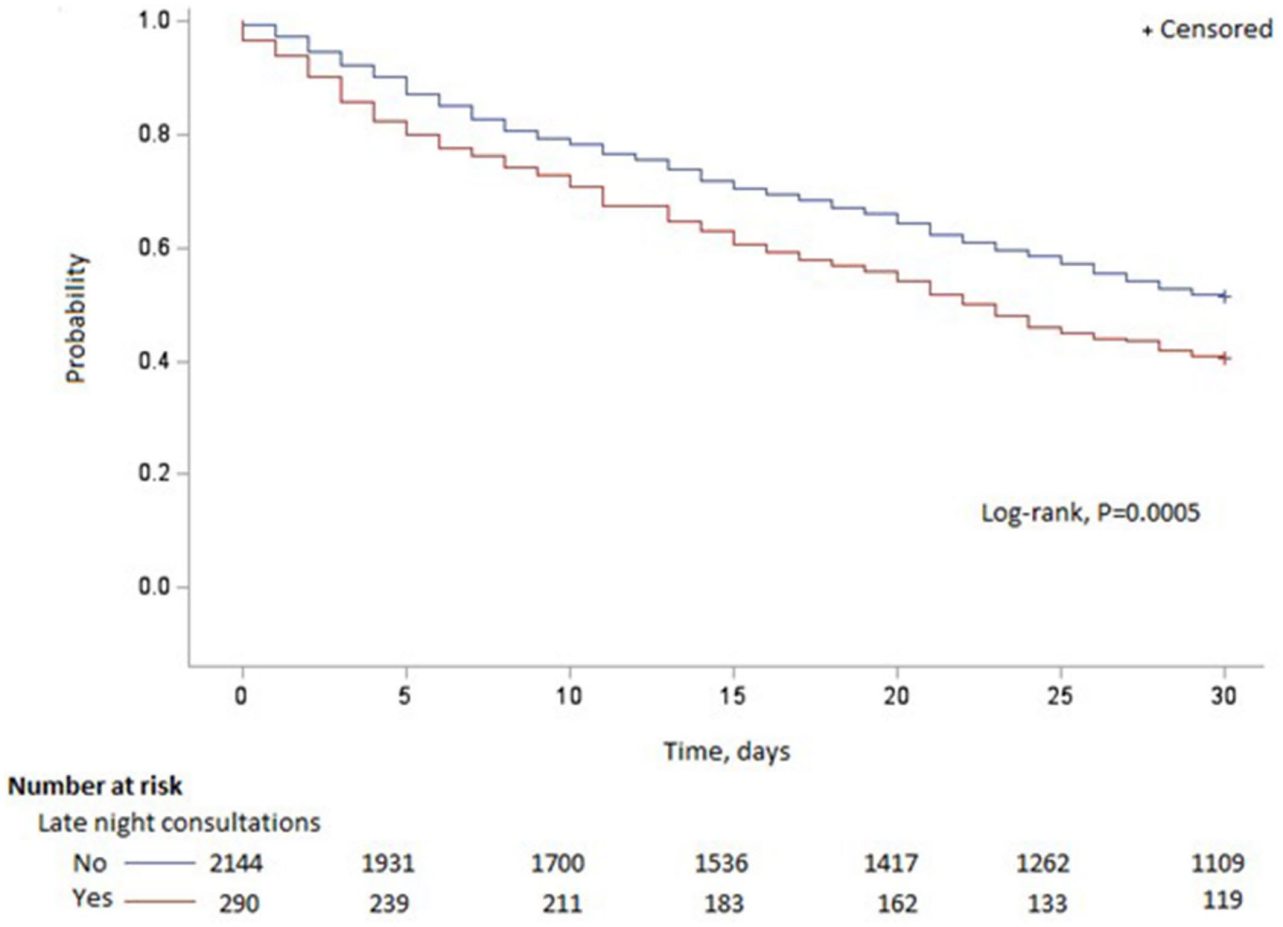
Distribution of reconsultations according to the initial late night consultation.

Table 3 presents the time to repeat consultation (in days). The risk factors “late night consultations” and “diagnosis = psychological disorders” were associated with significantly shorter time to repeat consultation, with HRs of 1.67 and 2.06, respectively. Figure 2 displays the time to repeat consultations among those whose initial consultation was held late at night, and those whose initial consultation was not late at night (midnight to 6.00 am).

**Table 3:** Factors associated with time to repeat consultation.

	Time to repeat consultation (days)	*p*-value	Adjusted HR^1^ (95%CI)	*p*-value
Location of the consultation		0.06		0.01
Home	30 (12–30)		1.00 (réf)	
At the surgery	28 (12–30)		1.29 (1.06–1.57)	
Time of the consultation				
Out of hours (6 pm-midnight, 6-8 am)		0.07		0.21
No	29 (11–30)		1.00 (réf)	
Yes	30 (16–30)		0.86 (0.68–1.09)	
Late night (midnight to 6.00 am)		0.0005		0.0002
No	30 (13–30)		1.00 (réf)	
Yes	22.5 (8–30)		1.67 (1.27–2.18)	
Weekend		<0.0001		<0.0001
No	28 (11–30)		1.00 (réf)	
Yes	30 (14–30)		0.68 (0.56–0.82)	
Motive for the consultation				
Acute problem		0.17	-	-
No	15 (5–30)			
Yes	30 (12–30)			
Main diagnosis				
Circulatory system		0.01		0.02
No	30 (12–30)		1.00 (réf)	
Yes	30 (22–30)		0.50 (0.27–0.91)	
Psychological		<0.0001		<0.0001
No	30 (12–30)		1.00 (réf)	
Yes	22.5 (9–30)		2.06 (1.46–2.91)	

^1^HR1 = variables entered in the model. HR = Hazard-ratio; CI, confidence interval. Variables included in the Cox proportional hazards regression with backward selection were location of the consultation, time of the consultation, main diagnosis, and matching as random effect.

The profile of patients aged >65 years did not differ from the profile of the overall population of patients.

## 4. Discussion

This study identifies two key features of unscheduled care consultations that are associated with an increased likelihood of repeat consultation within 30 days, namely, nighttime consulting (between midnight and 6.00 am) and the motive for consultation, as reflected by the main diagnosis, which were both associated with a significantly higher likelihood of repeat consultation, after matching for age and sex. These findings suggest that consultations held late at night are less efficacious. Second, consultations for psychological disorders also seem to give rise to more repeat consultations, suggesting that anxiety disorders are a major driver of healthcare consumption. Our findings suggest that consulting at the weekend is associated with a lower probability of re-consulting, possibly because more patients are addressed directly to the hospital at the weekend (in the absence of other available services), putting them on a different healthcare pathway and reducing the potential for repeat unscheduled consultations.

In the hospital setting, the number of unscheduled return visits or repeat consultations after attendance at the emergency department (ED) or surgery is widely used as an indicator of the quality of care (17–20). The rate of re-admission at 30 days has also been used as a quality indicator by Medicare and Medicaid centers in the United States (21–23). A number of studies have been performed to investigate the profiles of frequent users of healthcare resources, especially in the hospital setting (24). In a population-based, nationally representative study of over 45 million US adults with at least one ED visit, Hunt et al. (25) reported that the 8% of adults with four or more ED visits accounted for 28% of all consultations (24). There is a general consensus in the literature that frequent attenders usually present chronic diseases (3, 5, 26), and are predominantly young females, or persons aged over 65 (3, 5). Our finding that in primary care, unscheduled consultations with psychological disorders as the main diagnosis were associated with an increased likelihood of repeat consultation, is in line with previous reports from the ED setting. It is important to identify the profiles of frequent attenders in all healthcare settings, in order to enable adequate allocation of healthcare resources to cover the population’s needs. Therefore, there is potential for repeat consultation to be used as an indicator of quality of care in the primary care setting, as is the case in the ED setting.

The over-representation of psychological disorders as diagnoses in repeat consultations has been reported as a major risk factor for healthcare consumption, especially when associated with drug use (27). In France, in the ED, among patients who attend for medical problems, 10% reported that they came to the ED because of anxiety-related disorders (28). It has also been shown that among “super-users” of ED care (>18 ED visits per year), there is a core group who exhibited persistent frequent healthcare use over the long-term (up to 11 years) (29). An association between psychiatric disorders and sleep disorders has been widely documented, and night-time anxiety can exacerbate the psychiatric problems (30, 31). All of these findings are in line with our study, which showed that psychological disorders were independently associated with repeat consultation.

Among the few existing studies to have examined the characteristics of frequent users of ambulatory care and out-of-hours care (between 8 pm and 8 am), Buja et al. highlighted the prominence of psychiatric disease, but also socio-demographic factors in predicting frequent attendance at out-of-hours services (32). These authors also found that organizational factors also influenced frequent attender status, whereby frequent attender status is less likely when the GP works in a group practice setting (32). Finally, the meta-analysis by Foster et al. provides additional insights regarding the timing of the increased demand for unscheduled care, during the out-of-hours period (33). Specifically, they observed a peak in demand between 6 pm and 11 pm on weekdays. Saturday mornings have the highest level of activity of the weekend period, while consultations late at night (between midnight and 6.00 am) are more frequent at the weekend than during the week (33). These results are again congruent with our observation that night-time consultations are associated with an increased likelihood of repeat consultation. If we consider repeat consultation within 30 days as a quality indicator, it might be of value to implement prospective follow-up of this indicator over longer periods.

A third key point in our study is the fact that consulting at the weekend is associated with a lesser likelihood of a repeat consultation within 30 days. This appears to be in contradiction with existing literature, which describes a “weekend effect,” with reduced quality of care, and excess hospital mortality (34). A landmark study in this regard dates from 2001, when Bell et al. reported from a total of almost 3.8 million admissions in Ontario, Canada, that weekend admissions were associated with a significantly increased risk of mortality among patients with abdominal aortic aneurysm, acute epiglottitis, and pulmonary embolism (34). A large meta-analysis published from the United Kingdom including 97 studies totaling over 51 million patients, also reported higher mortality among patients hospitalized at the weekend, compared to those admitted on weekdays, with a relative risk of 1.19 (95%CI, 1.14–1.23) (35). The explanations for this weekend effect remain debated, and include a potentially lower number of staff at the weekend, staff with less experience, lack of access to invasive diagnostic or therapeutic procedures, or differences in severity of patients admitted at the weekend (36). However, the majority of these studies were performed in the hospital setting, and we could hypothesize that the increased hospital activity during these periods relieves some of the pressure on unscheduled primary care services.

In any case, in a context of the scarcity of physicians and the increasing volume of visits to unscheduled care services in the community, it is a major public health challenge to distribute existing resources in a manner that best meets patients’ needs. The strong presence of psychological disorders among the motives for consultation raises the question of the possible implementation of unscheduled care services specifically focused on mental health, or alternatively, wider access to mental health support services during daytime hours. Using repeat consultation within 30 days as a quality indicator could help to orient the type of management proposed in unscheduled care services, and/or quantify their contribution to primary care. Indeed, accessibility of care is not limited solely to the density of medical practitioners in a given area, and the geographical proximity of care services, but is multifactorial, with socio-economic factors of the patients also playing a role (37–40). This aspect represents an interesting avenue for future research into frequent healthcare use.

This study has some limitations. We focused on repeat consultations in general medicine, using unscheduled care services from a single provider, although we included a large number of consultations, which is a strength of the study. The study was retrospective, and its findings may not be generalizable to other patient populations. Furthermore, the measure of the primary outcome may be incomplete if some patients attended a repeat consultation in another healthcare structure, and this would have underestimated the repeat consultation rate in the present analysis. Lastly, there is potential for confusion bias, as we did not record patient comorbidities at baseline, and therefore, could not account for them in the analysis. Similarly, clinical data and prescriptions from home consultations are often insufficient to accurately analyze potential healthcare consumption between groups.

This study shows that patients who consult unscheduled primary care services have a higher likelihood of repeat consultation within 30 days if they present with psychological disorders, and if they consult late at night (between midnight and 6.00 am). These results suggest that the quality of care is not uniform and may vary, and therefore, patients consulting late at night may receive suboptimal care, and may be at risk of subsequently attending the emergency department. Improved monitoring of the quality of late-night primary care provision could have substantial public health repercussions, particularly if quality indicators were developed for surveillance and benchmarking of the quality of care of patients referred from primary care. A quality indicator based on re-consultation rates could be informative for orienting resources and identifying relevant patient care pathways.

Unscheduled care represents a key component of primary care provision in France, and is used by an increasing volume of patients, in view of the tension on hospital EDs. Understanding the patterns of healthcare use, and the profiles of the attenders at unscheduled primary care services, such as *SOS Médecins*, is of value in orienting the organization of primary care provision nationwide. Identifying a useful quality indicator could also be of use in monitoring quality of care in unscheduled care services. Our findings provide insights about the potential for improving access to mental health services.

## Data availability statement

The raw data supporting the conclusions of this article will be made available by the authors, without undue reservation. Requests to access the datasets and any queries can be directed to SS via stephane.sanchez@hcs-sante.fr.

## Author contributions

CR and SS were involved in the conception and design of the study. SS and VR were the coordinator of the study. VR were responsible for the data collection and wrote the first draft. LB was in charge of the analysis. VR, CR, and SS were involved in the interpretation, critically reviewed the first draft. All authors approved the final version and accept responsibility for the paper as published.

## Conflict of interest

The authors declare that the research was conducted in the absence of any commercial or financial relationships that could be construed as a potential conflict of interest.

## Publisher’s note

All claims expressed in this article are solely those of the authors and do not necessarily represent those of their affiliated organizations, or those of the publisher, the editors and the reviewers. Any product that may be evaluated in this article, or claim that may be made by its manufacturer, is not guaranteed or endorsed by the publisher.
